# Efficacy of TKIs in non-small cell lung cancer with atypical EGFR p.L747P and p.L747S mutations

**DOI:** 10.3389/fonc.2026.1717191

**Published:** 2026-01-27

**Authors:** Qiongxia Hu, Jing Wang, Juan Jiang, Zhujun Deng, Kang Xie, Wengeng Zhang, Weimin Li, Bojiang Chen

**Affiliations:** Precision Medicine Center, Precision Medicine Key Laboratory of Sichuan Province, West China Hospital, Sichuan University, Chengdu, Sichuan, China

**Keywords:** non-small cell lung cancer, p.L747P, p.L747S, tyrosine kinase inhibitor, uncommon EGFR mutation

## Abstract

**Objectives:**

Epidermal growth factor receptor (EGFR) tyrosine kinase inhibitors (TKIs) are established first-line treatments for advanced non-small cell lung cancer (NSCLC) harboring common sensitizing EGFR mutations, such as exon 19 deletions (19del) and the exon 21 p.L858R point mutation. However, evidence regarding the efficacy of first-, second-, and third-generation EGFR-TKIs against uncommon EGFR exon 19 mutations, specifically p.L747P and p.L747S, remains limited, and the underlying mechanisms are not fully elucidated. This study aimed to evaluate the clinical efficacy of different-generation EGFR-TKIs in NSCLC patients harboring EGFR p.L747P or p.L747S mutations by integrating our institutional cases with published evidence.

**Materials and methods:**

We identified patients with NSCLC harboring EGFR p.L747P or p.L747S mutations detected by next-generation sequencing (NGS) between 2020 and 2025 and retrospectively collected their clinical data. A literature review was conducted to identify and integrate relevant published case data.

**Results:**

Among seven treated stage IV NSCLC patients with the p.L747P mutation, two who received second-generation EGFR-TKIs as first-line therapy had an objective response rate (ORR) of 0% and experienced rapid disease progression. In contrast, six patients received third-generation EGFR-TKIs as first- or second-line therapy, with two achieving stable disease (SD) and one achieving partial response (PR), with a substantially longer progression-free survival (PFS) of 12 to 40 months. Separately, six patients were found to harbor the p.L747S mutation concomitantly with a common TKI-sensitive mutation, none of whom had received prior EGFR-TKI therapy. Of these, two patients were treated with a third-generation TKI: 1 achieved SD and the other achieved partial response (PR).

**Conclusions:**

This integrated retrospective analysis suggests that third-generation EGFR-TKIs may provide disease control (PR/SD) and prolonged PFS in a subset of NSCLC patients harboring uncommon EGFR p.L747P or p.L747S mutations, particularly those with co-existing sensitizing mutations or central nervous system metastases (CNS).

## Introduction

Lung cancer is the leading cause of cancer-related death worldwide. Non-small cell lung cancer (NSCLC) accounts for approximately 75-85% of all lung cancer cases (1). Epidermal growth factor receptor (EGFR) is the major driver oncogene in lung cancer, especially in Asian populations (2). Approximately 47-54% of patients diagnosed with advanced NSCLC in China harbor EGFR mutations (1). Short in-frame deletions in the amino acid ELREA of exon 19 (19del) and the exon 21 p.L858R point mutation are the most common alterations (3). Mutations in these regions change the spatial structure of the enzyme’s functional domain and lead to constitutive activation of EGFR and its downstream signaling activation (4).

EGFR mutations are oncogenic and alter the tyrosine kinase pocket of EGFR to a degree that enhances sensitivity to adenosine triphosphate (ATP)-competitive EGFR inhibitors. These factors increase the sensitivity of EGFR-mutated NSCLCs to EGFR tyrosine kinase inhibitors (TKIs) (5). The use of EGFR-TKIs has proven to be an effective therapy for advanced NSCLC with mutant EGFR (6–9). Patients with EGFR mutations, particularly those with exon 19 deletions (19del) or p.L858R mutations, are sensitive to TKIs (10). However, the characterization of rare EGFR mutations identified through gene sequencing remains insufficient (1, 4). Many uncommon EGFR mutations, such as p.G719X, p.S768I, and p.L861Q, affect approximately 10% of the NSCLC population (11–13), but little is known regarding their characteristics, activation, and sensitivity to various EGFR-TKIs, including allosteric inhibitors (2). Uncommon EGFR alterations appear to carry heterogeneous molecular features with clinically variable responses to TKIs and shorter progression-free survival (PFS) than common EGFR mutations do (14).

The p.L747P results from codon 747 of exon 19 with a two-base-pair (bp) mutation (c.2239_2240 TT>CC), leading to the replacement of leucine by proline (15). Similar to other activating common EGFR mutations, p.L747P drives the oncogenesis. However, a limited number of case reports have demonstrated that NSCLC patients with this mutation showed various responses to EGFR-TKI treatment. According to the structure-based classification by Robichaux et al., p.L747P and p.L747S mutations fall into the P-loop and αC-helix compressing (PACC) subgroup. This classification predicts that these mutations confer reduced efficacy to first- and third-generation TKIs, while making them particularly vulnerable to second-generation TKIs like afatinib (16). An analysis of the three-dimensional structure of the EGFR kinase revealed that Leu747 is located at the end of strand β3 connecting to the C-helix and some hydrophobic residues to stabilize the inactive function of the kinase (17). Theoretically, the substitutions of L747 with Pro or Ser with minimal structural changes, could cause constitutional activity of the EGFR kinase, which is sensitive to EGFR-TKI treatment (17, 18). However, in the literature, these published cases have reported heterogeneous responses to TKIs (10, 15, 18–26).

Although there is extensive clinical experience with EGFR inhibitors in common activating exon 19del and exon 21 p.L858R mutations, and more recently with exon 20 insertions, the efficacy against rare EGFR mutations is less clear. In such cases, clinicians must rely on preclinical studies, case reports, or small subsets from clinical trials for treatment guidance (26). The effectiveness of EGFR-TKIs in patients with these two rare point mutations in exon 19 of EGFR is seldom discussed. Therefore, a retrospective study was conducted on patients with either of these two uncommon mutations treated at West China Hospital of Sichuan University (WCH), supplemented by a review of published cases from PubMed. This study aimed to investigate the clinical characteristics and outcomes of these patients to evaluate the efficacy of EGFR-TKIs.

## Materials and methods

### Patient selection and data collection

We conducted a retrospective cohort study supplemented by a systematic literature review. Patients with NSCLC harboring EGFR p.L747P or p.L747S mutations, who were treated at WCH between October 2020 and October 2025, were identified through the institutional database. Clinical data, including demographics, treatment history, and imaging outcomes, were extracted from electronic medical records. For patients initially diagnosed elsewhere, formalin-fixed paraffin-embedded (FFPE) specimens were sent to the Department of Pathology at WCH for centralized analysis. All tumor samples underwent comprehensive genomic profiling via next-generation sequencing (NGS) using a panel covering 1021 cancer-related genes (Precision Medicine Center, WCH) to confirm EGFR mutation status. Intracranial response in patients with central nervous system (CNS) metastases was assessed via brain magnetic resonance imaging (MRI), per standard clinical protocols. The last follow-up date was November 30, 2025. Ethical approval was exempted for this retrospective study due to the use of de-identified patient data, in accordance with the guidelines of the Institutional Review Board of West China Hospital.

### Systematic literature review

A parallel systematic search of the PubMed database was conducted to identify published case reports or series describing NSCLC patients with EGFR p.L747P or p.L747S mutations. The search strategy employed the following key terms: “uncommon EGFR mutations,” “EGFR exon 19 mutations,” “EGFR p.L747P,” and “EGFR p.L747S.” Neither date nor language restrictions were imposed. Retrieved articles were screened based on titles and abstracts, and full texts of potentially eligible reports were reviewed. Studies were included if they reported on patients with histologically confirmed NSCLC harboring either the p.L747P or p.L747S EGFR mutation (exclusively or in combination with other EGFR mutations) and provided extractable data on clinical characteristics and/or treatment outcomes. Data from eligible publications were systematically extracted and integrated with our institutional cohort for comparative analysis (2, 5, 10, 19–33).

## Results

### Demographics of enrolled patients

From October 2020 to October 2025 at WCH, a total of 4079 patients were diagnosed with NSCLC, and 2159 (52.9%) patients tested positive for EGFR mutations by NGS. Among these EGFR-mutant patients, only 16 NSCLC patients (0.74%) carrying uncommon EGFR p.L747P (n=10) or p.L747S (n=6) mutations were identified and retrieved. The median patient age was 63 (range, 37-84) years. Thirteen patients (81.3%) were female, and 13 patients (81.3%) were never-smokers. Most patients (62.5%) were diagnosed at advanced stages, whereas 6 patients (37.5%) were diagnosed at early stages. The clinical, demographic, and molecular characteristics of patients harboring the EGFR p.L747P or p.L747S mutation from this cohort and published data are summarized in **Table 1**.

**Table 1:** Clinical characteristics of patients harboring EGFR p.L747P or p.L747S mutations in the WCH cohort and published cases.

Characteristic	p.L747P		p.L747S	
No. of patients	WCH cohort (n=10)	Published data (n=12)	WCH cohort (n=6)	Published data (n=9)
Age (years), median (range)	63 (41-84)	63.1 (41-84)	54.5 (37-66)	74.4 (62-82)
Sex, n (%)				
Male	2 (20.0)	3 (25.0)	1 (16.7)	2 (22.2)
Female	8 (80.0)	9 (75.0)	5 (83.3)	5 (55.6)
N/A	/	/	/	2 (22.2)
Smoking status, n (%)				
Current/ex-smoker	/	2 (16.7)	1 (16.7)	4 (44.4)
Never-smoker	9 (90.0)	6 (50.0)	4 (66.7)	2 (22.2)
N/A	1 (10.0)	4 (33.3)	1 (16.7)	3 (33.3)
Clinical stage, n (%)				
I/II	3 (30.0)	/	3 (50.0)	1 (11.1)
III	/	1 (8.3)	/	1 (11.1)
IV	7 (70.0)	9 (75.0)	3 (50.0)	2 (22.2)
N/A	/	2 (16.7)	/	5 (55.6)
Treatment in stage IV disease, n				
Third-generation EGFR-TKIs	7	4	3	2
First/second-generation EGFR-TKIs	3	17	/	6
Unspecified EGFR-TKIs	/	/	1	2

Data are presented as median (range) or n (%). Treatment data for stage IV disease represent counts of therapeutic regimens (lines of therapy), not unique patients; thus totals may exceed the number of stage IV patients due to sequential treatments (e.g., first-/second-generation TKI followed by third-generation TKI). First-/second-generation EGFR-TKIs included erlotinib, afatinib, gefitinib, and dacomitinib. Third-generation EGFR-TKIs included osimertinib, furmonertinib, and almonertinib.

EGFR, epidermal growth factor receptor; TKI, tyrosine kinase inhibitor; WCH, West China Hospital.

### Clinical outcomes

All 7 patients in this cohort from WCH, who had stage IV NSCLC harboring the p.L747P mutation, received EGFR-TKIs (**Table 2**). Two patients received second-generation EGFR-TKIs (afatinib) as first-line treatment. Neither of these 2 patients achieved an objective response, and both experienced progressive disease (PD). Among the 6 patients who received third-generation EGFR-TKIs as first- or second-line therapy, 2 achieved stable disease (SD) and 1 achieved partial response (PR), demonstrating a relatively long PFS ranging from 12 to over 40 months; notably, the patient with the longest PFS (>40 months) presented with brain metastases. However, 1 patient developed a rash within 30 days of receiving osimertinib, and after switching to almonertinib, the disease still progressed rapidly.

**Table 2:** Clinical characteristics and outcomes of 10 NSCLC patients with EGFR p.L747P mutations from WCH.

No.	Age/sex	Clinical stage	Smoking status	Year of Dx	EGFR mutation	TKI treatment (line)	Response to TKI	PFS (months)
1	46/F	IA	NS	2024	p.L747P	None	–	–
2	68/F	IB	NS	2022	p.L747P	None	–	–
3	75/M	IB	NS	2022	p.L747P	None	–	–
4	78/F	IVA	NS	2024	p.L747P	Almonertinib (1L)	PR	>14
5	57/F	IVA	NS	2017	p.L747P	Almonertinib (1L)	SD	>40
6	54/F	IVB	NS	2025	p.L747P	Furmonertinib (1L)	N/A	N/A
7	58/F	IVB	NS	2023	p.L747P	Afatinib (1L); Osimertinib (2L)	PD; PD	-; -
8	52/F	IVB	AS	2023	p.L747P	Osimertinib (1L); Almonertinib (2L)	NE; PD	-; -
9	63/M	IVB	NS	2008	p.L747P	Afatinib (1L)	PD	–
10	63/F	IVB	NS	2020	p.L747P	Gefitinib (1L); Osimertinib (2L)	PD; SD	-; 12

F, female; M, male; AS, active smoker; NS, never-smoker; Dx, diagnosis; 1L, first-line; 2L, second-line; EGFR, epidermal growth factor receptor; TKI, tyrosine kinase inhibitor; PFS, progression-free survival; PD, progressive disease; SD, stable disease; PR, partial response; N/A, not available.

Initial testing via amplification refractory mutation system PCR (ARMS-PCR) in Patient 10 detected an EGFR 19del but missed the p.L747P mutation, which was later revealed by NGS (20.6% abundance) after gefitinib failure. Given the presence of brain metastases, low systemic disease burden, and toxicity profile of alternative regimens, osimertinib was selected over the genomically recommended second-generation EGFR-TKI. The patient achieved sustained thoracic and intracranial responses on osimertinib at the 12-month follow-up.

The clinical characteristics of the 6 patients harboring the EGFR p.L747S mutation are summarized in **Table 3**. In our study, the p.L747S mutation, previously documented to confer resistance to EGFR-TKIs, was identified in only 6 individuals. All of these patients carried concurrent TKI-sensitive mutations (e.g., 19del, p.L858R, or p.G719C), and none had received EGFR-TKI treatment prior to NGS testing. Of these, only the 2 patients with stage IVB disease received third-generation EGFR-TKIs: Patient 5 (with brain metastases) achieved a PR on osimertinib with a PFS of >9 months. Patient 4 achieved SD on furmonertinib (PFS 5 months), was switched to almonertinib due to hematologic toxicity, and maintained SD for a further 8 months with ongoing response at the last follow-up. The remaining 4 early-stage patients did not receive TKI therapy.

**Table 3:** Clinical characteristics and outcomes of 6 NSCLC patients with EGFR p.L747S mutations from WCH.

No.	Age/sex	Clinical stage	Smoking status	Year of Dx	EGFR mutation (compound)	TKI treatment (line)	Response to TKI	PFS (months)
1	43/F	IA	NS	2022	p.L747S-p.T751_A755del	None	–	–
2	37/F	IA	NS	2022	p.L747S-p.L858R	None	–	–
3	60/F	IIB	NS	2021	p.L747S-p.L747_P753delinsS	None	–	–
4	55/F	IVB	AS	2024	p.L747S-p.T751_A755del	Furmonertinib (1L); Almonertinib (2L)	SD; SD	5; 8
5	66/F	IVB	NS	2024	p.L747S-p.L858R	Osimertinib (1L)	PR	>9
6	65/M	IVB	AS	2025	p.L747S-p.G719C	None	–	–

F, female; M, male; AS, active smoker; NS, never-smoker; Dx, diagnosis; 1L, first-line; 2L, second-line; EGFR, epidermal growth factor receptor; TKI, tyrosine kinase inhibitor; PFS, progression-free survival; SD, stable disease; PR, partial response; N/A, not available.

Reviewing the published literature, we identified 21 patients harboring p.L747P (n=12) or p.L747S (n=9) mutations. The following analysis focuses on the 12 patients with p.L747P, whose data were integrated into our study (**Table 1**). The median age of the cohort was 63.1 years (range, 41-84). Nine patients (75.0%) were female and 6 (50.0%) were never-smokers. Regarding disease stage, 9 (75.0%) had stage IV lung adenocarcinoma, 1 (8.3%) had stage IIIA disease, and staging information was unavailable for 2 (16.7%). All 12 patients received EGFR-TKIs, including gefitinib (n=7), afatinib (n=6), osimertinib (n=4), erlotinib (n=3), and dacomitinib (n=1). As some patients received multiple lines of therapy, the total number of treatment instances exceeds the patient number. Treatment responses are summarized in **Table 4**.

**Table 4:** Clinical characteristics of 12 published lung adenocarcinoma patients harboring the EGFR p.L747P mutation.

No	Age/sex	Clinical stage	Smoking status	Year of report	EGFR mutation	EGFR-TKIs	Response to TKI	PFS (months)	Testing	Reference
1	61/M	IV	AS	2016	p.L747P	Erlotinib	PD	–	Sanger sequencing	(21)
2	80/F	IV	NS	2020	p.L747P	Gefitinib; Osimertinib	SD; SD	18; >4	NGS	(25)
3	84/M	IV	AS	2022	p.L747P	Osimertinib	SD	7	NGS	(26)
4	66/F	IIIA	NS	2022	p.L747P	Afatinib; Dacomitinib	SD; PR	7; 17	NGS	(23)
5	76/F	IV	NS	2015	p.L747P-p.E746V	Gefitinib	PD	–	Sanger sequencing	(34)
6	44/F	IV	NS	2018	p.L747P-p.R521K	Afatinib	SD	>24	NGS	(19)
7	54/F	IV	NS	2018	p.L747P	Gefitinib; Afatinib; Erlotinib; Osimertinib	PD; NA; SD; PD	-; 7; 7; -	NGS	(10)
8	66/M	IV	N/A	2015	p.L747P	Gefitinib	PD	–	Sanger sequencing	(20)
9	69/F	N/A	N/A	2021	p.L747P	Gefitinib; Afatinib	SD; PR	4; 4	NGS	(2)
10	57/F	IV	N/A	2022	p.L747P	Afatinib; Osimertinib; Erlotinib	PD; SD; OS	-; 36; >96	NGS	(22)
11	59/F	IV	N/A	2012	p.L747P	Gefitinib	SD	6	Sanger sequencing	(27)
12	41/F	N/A	NS	2022	p.L747P	Gefitinib; Afatinib	PD; SD	-; 4	NGS	(24)

F, female; M, male; AS, active smoker; NS, never-smoker; EGFR, epidermal growth factor receptor; TKI, tyrosine kinase inhibitor; NGS, next-generation sequencing; PFS, progression-free survival; PD, progressive disease; SD, stable disease; PR, partial response; OS, overall survival; N/A, not available; NA, not available.

Notably, initial testing via PCR-based methods misclassified the mutations in patients 2, 9, and 12 as common EGFR 19del, whereas subsequent NGS correctly identified these variants as p.L747P. Patient 12, who received gefitinib, exhibited PD, showing a treatment response similar to that of Patient 10 in our cohort. Analysis of TKI efficacy across different generations revealed heterogeneous outcomes. Among those treated with first-generation TKIs, 4 who received gefitinib showed resistance with PD while 3 achieved SD, with PFS ranging from 4 to 18 months. Of the 6 patients treated with second-generation TKIs, 1 who received afatinib achieved a PR (PFS of 4 months), 3 achieved SD on afatinib with PFS ranging from 4 to 24 months, and 1 had PD. Notably, 1 patient achieved SD for 7 months with afatinib and subsequently achieved a PR with a PFS of 17 months after switching to dacomitinib. Regarding third-generation TKIs, 3 patients with the p.L747P mutation who received osimertinib achieved a PFS ranging from 4 to 36 months, whereas 1 patient failed to respond, with a PFS of only 1 month.

The EGFR p.L747S mutation was identified in 9 patients, with detailed information extracted from published studies summarized in **Table 5**. Their ages ranged from 62 to 82 years, with 5 males and 4 reporting a smoking history. Age and gender data were unavailable for 2 patients. Among them, 6 received specified EGFR-TKIs: 3 with first-generation (erlotinib or gefitinib), 1 with second-generation (afatinib), and 2 with third-generation (osimertinib). The overall treatment outcomes were as follows: 1 patient achieved a PR, and the rest maintained SD. The 3 patients on first-generation TKIs had a PFS of 6–48 months. The patient receiving afatinib achieved SD with a PFS of 6 months, while the 2 on osimertinib achieved a PFS of 12 and 16 months, respectively.

**Table 5:** Clinical characteristics of 9 NSCLC patients harboring the EGFR p.L747S mutation in the published literature.

No	Age/sex	Clinical stage	Smoking status	Year of report	EGFR mutation	EGFR-TKIs	Response to TKI	PFS (months)	Testing	Reference
1	81/M	N/A	AS	2024	p.L747S-p.G719C	Osimertinib	SD	16	NGS	(28)
2	71/F	IVA	NS	2019	p.L747S-p.G719C	Osimertinib	SD	12	Targeted RNA sequencing	(29)
3	N/A	N/A	N/A	2018	p.L747S	Erlotinib	SD	>48	N/A	(30)
4	62/M	N/A	N/A	2020	p.L747S-p.G719S	Afatinib	SD	6	NGS	(31)
5	N/A	N/A	N/A	2008	p.L747S-p.L858R	Gefitinib; Erlotinib	SD; PR	40; 6	N/A	(32)
6	78/M	IB	AS	2014	p.L747S-p.G719S	N/A	N/A	N/A	direct-sequenced	(33)
7	73/M	IV	AS	2014	p.L747S	N/A	N/A	N/A	direct-sequenced	(33)
8	82/M	IIIB	AS	2014	p.L747S	N/A	N/A	N/A	direct-sequenced	(33)
9	74/F	N/A	NS	2008	p.L747S-p.L858R	Gefitinib; Erlotinib	N/A; SD	N/A; 6	sequenced	(5)

F, female; M, male; AS, active smoker; NS, never-smoker; 1L, first-line; 2L, second-line; EGFR, epidermal growth factor receptor; TKI, tyrosine kinase inhibitor; PFS, progression-free survival; PD, progressive disease; SD, stable disease; PR, partial response; N/A, not available.

Published studies indicate that the p.L747S mutation can emerge as a resistance mechanism to prior gefitinib therapy, co-occurring with the original p.L858R mutation (5, 32). Specifically, 2 patients developed the secondary p.L747S mutation alongside the activating p.L858R mutation after treatment with gefitinib. These 2 patients (cases 5 and 9) carrying the p.L858R-L747S mutations achieved a partial radiographic response to erlotinib (150 mg/day) that lasted for 6 months.

## Discussion

Due to the rarity of the p.L747P and p.L747S mutations in NSCLC, their precise incidence remains challenging to determine. A cohort study from Taiwan, China, identified only 12 cases among 2,031 EGFR-mutant LUAD patients, corresponding to an incidence of approximately 0.59% (18). In our study, 16 patients (0.74%) harbored these uncommon mutations, confirming their low prevalence in the EGFR-mutant population. Current evidence suggests that the EGFR p.L747P mutation is generally associated with intrinsic resistance to first- (e.g., erlotinib, gefitinib) and third-generation (e.g., osimertinib) EGFR inhibitors, while exhibiting greater sensitivity to second-generation (e.g., afatinib, dacomitinib) EGFR-TKIs (23, 26). Using a patient-derived xenograft model with the p.L747P mutation, Yang et al. predicted that second-generation TKIs have the strongest binding affinity to the p.L747P mutant protein. Cellular kinase inhibition assays and xenograft experiments further confirmed that afatinib potently inhibits p.L747P-mutant cells, significantly suppresses tumor growth (P<0.001), and reduces phosphorylation of EGFR and its downstream signaling pathways (15). Recent reports also indicate that afatinib demonstrates superior activity compared to other EGFR-TKIs in patients with p.L747P or p.L747S mutations (18, 35). In a study by Moran et al., 1 patient with the p.L747P mutation achieved a PR as the best response, received afatinib for 10 months, and was still on treatment at data cutoff. Another patient with the p.L747S mutation also achieved a PR, was treated with afatinib for 4.1 months, and continued to receive the drug at data cutoff (36). Similarly, Li et al. reported a patient carrying p.L747P who was treated with later-line dacomitinib and achieved partial remission, with a PFS of 17 months (23).

In contrast to these reports emphasizing second-generation TKI efficacy, and contrary to the structure-based prediction by Robichaux et al. which classifies p.L747P/p.L747S into a subgroup (PACC) with predicted sensitivity to second-generation TKIs (16), our clinical observations present a divergent narrative. In our cohort, none of the patients who received first- or second-generation TKIs achieved a tumor response. However, half of those treated with third-generation TKIs attained either a PR (lasting over 14 months) or SD (ranging from 12 to 40 months). This apparent discrepancy between preclinical prediction, prior case reports, and our findings necessitates careful interpretation within the specific context of our study. First, our cohort’s sample size is limited, and the observed benefit with third-generation TKIs was primarily in the form of disease stabilization. Second, and critically, our patient population exhibited considerable heterogeneity. It included individuals across different disease stages (early to advanced), with varying EGFR mutation profiles (isolated p.L747P/p.L747S vs. compound mutations coexisting with classic sensitizing alterations such as exon 19del or p.L858R), and diverse treatment histories (ranging from TKI-naïve to multiple prior lines). This heterogeneity, particularly the presence of co-existing sensitizing mutations in all p.L747S cases, likely constitutes a major confounding factor, potentially altering overall kinase dynamics and drug response, thereby limiting direct comparability of outcomes across different TKI classes. We hypothesize that these compound genotypes, rather than the isolated p.L747P/p.L747S mutation alone, may be a key determinant of the clinical outcomes observed with third-generation TKIs in our series. It is essential to distinguish this clinical observation from the underlying molecular mechanism, which remains speculative without functional validation.

Resistance to EGFR-TKIs is categorized as either primary or acquired. Primary resistance denotes the immediate ineffectiveness of therapy, while acquired resistance refers to disease progression following a period of clinical benefit. Although acquired resistance in advanced NSCLC patients with sensitive EGFR mutations is well-characterized, understanding of primary resistance remains limited (10, 37, 38). The p.L747S mutation is a rare alteration previously reported to confer TKI resistance, a finding supported by preclinical evidence (30). For instance, Costa et al. demonstrated that Ba/F3 cell lines expressing the p.L858R-p.L747S double mutation exhibit intermediate resistance to gefitinib, suggesting that p.L747S may attenuate drug-induced apoptosis (39). This preclinical finding of resistance for the p.L858R-p.L747S double mutant intriguingly contrasts with the clinical benefit we observed in our p.L747S patients (who also had co-existing sensitizing mutations) treated with third-generation TKIs, again highlighting the complex translation from model systems to patient outcomes. However, clinical outcomes can be complex. Among our 6 patients with p.L747S, 2 received third-generation TKI: 1 achieved SD and the other had a PR. This aligns with the findings of Chiba M et al., who reported that cell lines with uncommon secondary mutations like p.L858R-p.L747S retain sensitivity to irreversible EGFR-TKIs (40). Furthermore, Swami U et al. suggest that patients with coexisting TKI-sensitive and -resistant mutations may benefit from TKI therapy, potentially achieving extended survival, possibly through strategies such as dose escalation (30).

Notably, a substantial proportion of uncommon EGFR mutations cannot be detected by the PCR-based methods commonly used in clinical practice. Furthermore, variables such as tumor sample adequacy, quality, and heterogeneity further complicate these detection techniques. The combination of these factors results in inaccuracies and biases in the reported incidence of less common EGFR mutations (41). With these methods, the p.L747P mutation may be incorrectly identified as 19del or false-negative as wild type, resulting in incorrect information for the guidance of clinical management (23, 42, 43). Given the urgent need for more comprehensive genetic profiling in advanced NSCLC, the clinical introduction of NGS with broad gene panels has significantly improved the detection of uncommon EGFR alterations and enables accurate characterization of EGFR mutation status (44, 45). Therefore, for patients treated with a first-generation EGFR-TKI for a reported 19del, who develop primary resistance, NGS can be used to re-evaluate these cases, providing critical information for more personalized therapy (18). With the increasing clinical use of NGS, the identification of patients who carry the p.L747P mutation but were initially diagnosed with EGFR exon 19 deletion is expected to become more common (2).

Several key limitations of this study must be acknowledged. First, its retrospective nature, small sample size, and inherent biases associated with the literature review restrict the generalizability of the findings. Moreover, the pronounced clinical and molecular heterogeneity within our cohort-spanning diverse disease stages, EGFR mutation profiles (isolated versus compound), and prior treatment lines-hinders direct outcome comparison and complicates the attribution of efficacy to any specific TKI generation. Second, while potential mechanisms are proposed to explain the observed sensitivity to third-generation TKIs, such explanations remain speculative without functional validation. Therefore, future research should integrate two essential approaches: well-designed prospective studies that include larger, molecularly characterized cohorts with stratified analyses, and functional experiments using appropriate models to clarify the underlying biology and drug sensitivity of these rare EGFR variants.

## Conclusions

NGS is recommended for accurate detection of rare EGFR p.L747P/p.L747S mutations, as PCR may misclassify them as 19del or wild-type. Third-generation EGFR-TKIs may provide modest disease control (SD/PR) and prolonged PFS in select patients with these mutations, particularly those with compound EGFR alterations or CNS metastases. However, these findings are preliminary due to small sample size and heterogeneity, and conclusions regarding “superior efficacy” should be viewed cautiously. Future prospective studies with larger cohorts and functional validation are needed to clarify the optimal TKI for this patient population.

## Data Availability

The raw data supporting the conclusions of this article will be made available by the authors, without undue reservation.
